# Prevalence and Associated Factors of Dental Caries in Primary Schoolchildren: An Iranian Setting

**DOI:** 10.1155/2020/8731486

**Published:** 2020-01-21

**Authors:** Mohammad Ali Youssefi, Solaiman Afroughi

**Affiliations:** ^1^Department of Endodontic, Yasuj University of Medical Sciences, Yasuj, Iran; ^2^Department of Biostatistics and Epidemiology, Yasuj University of Medical Sciences, Yasuj, Iran

## Abstract

**Introduction:**

Dental caries is the most common oral health disease of school-aged children around the world. In this study, we aimed to assess the prevalence and associated factors of dental caries in primary schoolchildren in Yasuj township, Iran.

**Methods:**

In this cross-sectional study, a total of 460 children aged 7–12 years were investigated. Dental examination was performed at school according to the World Health Organization criteria. Sociodemographic data were collected using a structured questionnaire, and caries statuses of children's teeth were recorded through a dental chart. Data were analyzed using summary statistics, chi-square test, and logistic regression model with odds ratio.

**Results:**

The prevalence of dental caries in primary, permanent, and whole dentition among children was 75.3%, 41.1%, and 89.8%, respectively. Among all considered factors, the caries presence in primary teeth was inversely (*p* < 0.001) and in permanent teeth was positively (*p* < 0.001) associated with the children's age. Moreover, the odds of decaying permanent teeth were significantly higher in girls, in rural children, and in children whose fathers were not an employee compared to their counterparts (*p*=0.04, *p* < 0.001, and *p*=0.02, respectively).

**Conclusions:**

The prevalence of dental caries among the studied primary schoolchildren in mixed dentition was high and associated with their sociodemographic factors. Providing and implementing preventive, therapeutic, and informative programs for controlling dental caries at individual, family, and school levels are necessary for local health policymakers.

## 1. Introduction

Dental caries is the most prevalent disease of the oral health in school-aged children around the world [1–5]. Dental caries leads to tooth pain, discomfort, eating impairment, loss of tooth, and delayed language development in children [6–8]. Furthermore, dental caries affects children's functions and body growth and imposes a financial burden on their families [2, 3, 9]. In addition, children with dental caries are exposing to fear and anxiety which can result to both severity and incomplete treatment of the condition [10].

According to World Health Organization reports, dental caries affects 60–90% of schoolchildren in both developing and developed countries [11, 12]. Furthermore, studies have shown that the prevalence of dental caries was up to 83.3% among Middle-Eastern schoolchildren [13–15].

Considering that school age is an influential period during which every child extends health related behaviors, beliefs, and attitudes and that the disease is irreversible, efforts should be focused on revealing factors that predispose/resist students to dental caries during this stage [1, 16–21]. An extent spectrum of factors influencing this disease has been documented in the literature [17, 18, 22]. However, because socioeconomic factors have the possibility to influence the prevalence of dental caries in children through their effects on oral health practices and parental oral health knowledge and attitudes as well as health care accessibility, recently, they have concerned the investigators [23]. Studies have reported that children's age and sex and parents' education, occupation, and residential place have an impact on their dental caries experiences [2, 18, 24]. However, data on the association of parents' occupation and resident place with dental caries in children are limited [9, 23].

In Iran, a Middle-Eastern country, according to several studies, the prevalence of dental caries among children compared with the developed countries was high [13, 25, 26]. However, evidence on the prevalence of caries in the mixed dentition of children is scarce [25, 27]. Additionally, in this country, no recent studies were found which simultaneously targeted the prevalence of tooth caries among primary schoolchildren in all age groups and in both urban and rural settings [13, 27].

Furthermore, at present, little is known about how sociodemographic factors influence the presence of caries in primary schoolchildren during the mixed dentition stage, especially in the region of this study [28, 29]. However, providing data on the burden and related factors of dental caries among primary schoolchildren could help health policy decision-makers to plan preventive and therapeutic programs.

Therefore, the aim of this study was to evaluate the prevalence of dental caries and to identify the association of factors, including the age, gender, family's educational level, employment status, and residential place, with the caries experience in 7–12-year-old primary schoolchildren in Yasuj township, Iran.

## 2. Methods

### 2.1. Study Design and Setting

The present analytical cross-sectional study was conducted from October 2013 to January 2014 among primary schoolchildren at Yasuj township, Iran.

### 2.2. Study Participants

Primary schoolchildren, aged 7–12 years at grades 1–5 (a grade or degree is a one-year education course), who were living in Yasuj township were included in the study. With regard to an estimate from a previous survey as 85%, a 95% confidence interval, and an error of 5% below or above the estimated value and using the single population proportion formula, the calculated sample size was 460 children. A three-stage stratified cluster random sampling technique was applied to select the study participants.

### 2.3. Data Collection and Dental Examination

Sociodemographic characteristics, such as the child's sex (male/female), age (7, 8, 9, 10, 11, and 12 year olds), school grade during the study (1–5), residence area (living in a village belonging to Yasuj township was considered to be rural, and living in Yasuj city was considered to be urban), parents' education level (primary, high school, complete high school, and bachelor's or higher), and parents' professional situation (office worker, freelancer, and/or housewife) (employee/not employed), were collected using a structured questionnaire. Dental examination was performed at the schools for all selected children by two professionals and calibrated dentists (kappa = 85%) according to the World Health Organization (WHO) dental caries diagnosis guideline. After that, the numbers of decayed teeth, teeth missing due to caries, and filed teeth for primary teeth (dmft) and permanent teeth (DMFT) were obtained using a dental chart (table). Finally, the dmft/DMFT index was implemented to describe children's caries experience.

### 2.4. Statistical Analysis

Statistical analysis was performed using the software Statistical Package for Social Sciences, version 24 (SPSS, Inc., Chicago, Illinois, USA). For each student, dental caries was experienced in primary teeth as dmft > 0 and in permanent teeth as DMFT > 0. Wholly, the caries prevalence (%) of each dentition system was calculated as the sum of the children affected by caries (for whom dmft > 0 or DMFT > 0) divided by the total number of participated children multiplied by 100. For each group, the prevalence of dental caries was computed as the number of affected children divided by group size.

Descriptive statistics were employed to compute the mean and standard deviation of quantitative variables. Frequencies (numbers and proportions) were implemented to assess prevalence of dental caries among groups. Contingency tables with the chi-square (*χ*^2^) test or equivalently proportion test were used for bivariate analyses of dependent variable and independent categorical variables. Univariate logistic regression analysis was implemented to detect the association of independent or explanatory variables with the dependent variable. The socioeconomic characteristics were considered to be independent variables, and dental caries (present, not present) in children was considered to be the dependent or outcome variable. Association assessments due to primary teeth and permanent teeth were separately accomplished. The crude odds ratio (OR) with its 95% confidence interval was applied to assess the strength of the associations. The significance level was set at *p*-value (*p* < 0.05).

### 2.5. Ethical Considerations

The study was approved by the research committee of Yasuj University of Medical Sciences, Iran. Before the survey, informed consent forms completed by children' parents were obtained. Cases with dental caries were referred to visit a dentist.

## 3. Results

All the children who received and returned the consent forms attended the survey, so the respond rate was 100%. The sociodemographic characteristics of the studied children and their parents are shown in Table 1. The prevalence of dental caries in the surveyed children according to sociodemographic variables and dentition type is displayed in Table 2. The caries prevalence of primary teeth, permanent teeth, and whole dentition (mixed of primary and permanent teeth) among children was 75.3%, 41.1%, and 89.8%, respectively.

There were significant differences in the prevalence of dental caries in the three age groups in both primary (*x*^2^ = 100.1, *p* < 0.001) and permanent (*x*^2^ = 27.3, *p* < 0.001) teeth. Considering the children's sex, the caries prevalence of primary teeth in boys was not significantly more than that of girls (*p*=0.42); however, the caries prevalence of permanent teeth in girls was significantly different from that in boys (*x*^2^ = 4.1, *p*=0.04). Although the findings indicate that the caries prevalence of primary teeth in urban children was not significantly higher than that in rural residents, the caries prevalence of permanent teeth between rural and urban children was significantly different (*x*^2^ = 20.83, *p* ≤ 0.001). In addition, while the caries prevalence of primary teeth respective to father's occupation did not show a significant difference (*p*=0.13), the caries prevalence of permanent teeth in the children whose fathers were not an employee was significantly greater than that of children whose fathers were an employee (*p*=0.028). Furthermore, neither in the primary dentition nor in the permanent dentition, caries prevalence was significantly different in terms of the mother's occupation conditions (*p*=0.84 and *p*=0.34, respectively) and mother's education status (*p*=0.72 and 0.54, respectively).

Output of the univariate logistic regression analysis (Table 3) showed that the children's age was significantly associated with the caries presence of both primary and permanent teeth (*p* < 0.001 and *p* < 0.001, respectively). The odds of decaying permanent teeth of children in the age groups 910 years and 1112 years were significantly higher than that of children in the age group 7–8 years. Vice versa, the probability of caries experience of primary teeth of children in the age groups 9–10 years and 11–12 years were significantly lower than that of children in the age group 7–8 years. Furthermore, in permanent teeth, caries presence was significantly associated with the sex, residential place, and father's occupation (*p*=0.04, *p* < 0.001, and *p*=0.02, respectively) in children. However, in primary teeth, there was not found any significant association between caries presence and sex, residential place, father's occupation and education, and mother's occupation and education. In addition, in permanent teeth, caries presence was not significantly associated with the father's education, mother's education and mother's occupation among children (Table 3).

## 4. Discussion

Most of the carried out studies have investigated the tooth caries among children at the two age groups: less than or equal six and 12 or higher years. The present study has provided data on the prevalence and associated factors of dental caries at mixed dentition stage among 7–12-year-old schoolchildren. The caries prevalence of primary, permanent, and whole dentition obtained in this study were much higher than the criteria set by the WHO [30] and the values presented in similar age groups of children in developed countries [21, 31–34]. Moreover, the caries prevalence found in this study resulted higher than those reported in several studies carried out in other Asian countries such as Yemen [15], India [20], Malaysia [35], and China [36]. These findings may be explained as below. First, the dental caries is a major oral health problem in the studied primary schoolchildren. Second, the oral health care system in Iran is not sufficiently developed, and the cost of dental caries treatment is very expensive [26]. Finally, disparity in intake habits, cultural status, and levels of parents' oral health knowledge, attitude, and practices among children of the aforementioned countries, compared with Iranian, might influence the differences [37].

In assessing the characteristics and factors associated with the dental caries, it was shown that as age increased, caries prevalence of primary teeth among children significantly decreased. Also, as it was demonstrated, caries prevalence of primary teeth was high. This may be due to lack of awareness about health caring and retaining of primary teeth as well as the parental attitude that the primary teeth are exchangeable by permanent teeth and are not important [21, 38, 39]. Consequently, it has been led to early exfoliation and/or extraction of the teeth which resulted in reduced number of primary teeth at older ages [36, 38]. This result is in agreement with findings reported in studies accomplished by Joshi et al. [22], Yabao et al. [38], Wu et al. [40], and Gatou et al. [37]. Besides, via both bivariate and logistic regression analysis, it was revealed that the caries presence of permanent teeth was significantly associated with the children's age in a direct manner. As was previously stated, early loosing of the primary teeth could result in early eruption of the permanent teeth. Therefore, at older ages, the permanent teeth were more exposed to factors influencing caries presence [36]. Another reason could be attributed to the inappropriateness of oral hygiene practices among studied children [22, 37, 38]. This is in accordance with the results of numerous published studies including Dawkins et al. [8], Veiga et al. [2], Wulaerhan et al. [6], Gatou et al. [37], Ditmyer et al. [41], and Martins et al. [42]. Additionally, the caries presence of permanent teeth was associated with the children's sex, such that its prevalence in the girls was significantly higher than that in the boys. This may be attributed to the permanent teeth that erupt earlier in girls than boys. Therefore, being exposed to influent factors for longer time could raise the caries prevalence of permanent teeth among the girls [43]. In addition, as previous studies reported, intake behaviors, hormonal fluctuations during puberty, social role in family and combination, and flow rate of saliva between boys and girls are different [43, 44]. All these dissimilarities might expose girls to be more susceptible to dental caries presence than boys. This result agrees with the results of a national study by Jessri et al. [13], studies by Heinrich-Weltzien et al. [45] in the Philippines and Zhang et al. [3] in Hong Kong, and a study by Laganà et al. [46] in Albania.

In the present study, as results show the caries prevalence of permanent teeth had an association with the residence place of the children, such that the odds of decaying permanent teeth of the rural children were notably higher than those of the children living in urban setting. In Iran, dentistry clinical services have not been yet established in rural places [26] and rural children for using dentistry services have to refer to urban facilities and to bear exhaust costs. Therefore, these children had no suitable accessibility to the dentistry heath care system and facilities. Furthermore, the observed difference of dental caries may be resulted from differences of socioeconomic factors, intake behaviors, and lifestyle conditions between the rural and urban communities. However, the presented result is supported by Dawkins et al. [8], Jürgensen and Petersen [7], Veiga et al. [2] Zemaitiene et al. [44], and Al-Darwish et al. [43].

As results demonstrated caries presence of permanent teeth among children was associated with the father's occupation, such that the likelihood of caries experience in children whose fathers were not an employee was higher than peers. Several studies have revealed that the socioeconomic conditions of parents are correlated with dental caries experiences of children [14, 15]. Iranian employees, especially those who live in the studied region, due to stable income through receiving salary, belong to medium- to high-level social category, proportionately. Families with higher income could better provide and have more accessibility to oral health care instruments and treatment needs compared with lower income families.

The present study had some limitations which addressing them could be considered in future studies. The detection of dental caries presence in the children was performed without taking radiography. Furthermore, the associated factors of dental caries in children could be better detected and evaluated in a longitudinal study.

## 5. Conclusion

The prevalence of dental caries in mixed state among primary schoolchildren obtained in the present study compared to WHO targets, developed countries, some developing countries, and national mean rates was high. In both primary and permanent teeth, caries presence was associated with the children's age, while in the permanent teeth, sex, residence place, and occupation of the father were associated with the caries presence among the children. Younger children were at a higher risk of experiencing caries of primary teeth, whereas older children, female children, children residing in rural areas, and children with fathers not an employee were at a higher risk of experiencing caries of permanent teeth. Therefore, providing and implementing preventive, therapeutic, and informative programs for controlling dental caries at individual, family, and school levels are necessary for local health policymakers.

## Figures and Tables

**Table 1:** Sociodemographic characteristics of the participated children and their parents.

Characteristics	Children (*n*)	%	Mean (sd)
Total sample	460	100	

Gender			
Girl	215	46.7	
Boy	245	53.3	

Age group (years)	—	—	9.5 (1.55)
7–8	133	28.9	7.6 (0.5)
9–10	179	38.9	9.4 (0.49)
11–12	148	32.2	11.3 (0.48)

Residence area			
Rural	154	33.5	
Urban	306	66.5	

Father's occupation			
Employee	189	41.1	
Not employee	270	53.7	

Mother's occupation			
Housewife	398	86.5	
Not housewife	62	13.5	

Father's educational level			
Under high school	82	17.8	
Complete high school	140	30.4	
University	197	42.8	

Mother's education level			
Under high school	134	29.1	
Complete high school	194	42.2	
University	90	19.6	

**Table 2:** Prevalence of caries according to sociodemographic variables and dentition type among children (*n* = 460).

Variable	Children (*n*)	Primary teeth		Permanent teeth	
		Caries, *n* (%)	*p* value^*∗*^	Caries, *n* (%)	*p* value^*∗*^
Age (year)			<0.001		<0.001
7–8	133	126 (94.7)		35 26.3	
9–10	179	151 (84.4)		70 (39.1)	
11–12	148	69 (46.6)		84 (56.8)	
Total	460	346 (75.2)		189 (41.1)	

Sex			0.42		0.04
Girl	215	158 (73.5)		99 (46)	
Boy	245	188 (76.7)		90 (36.7)	

Residence area			0.79		<0.001
Rural	154	229 (74.8)		86 (55.89)	
Urban	306	117 (76)		103 (33.7)	

Father's occupation			0.13		0.028
Employee	189	149 (78.8)		66 (34.9)	
Not employee	270	196 (72.6)		122 (45.2)	

Father's education			0.74		0.05
Under complete high school	82	61 (74.4)		42 (51.2)	
Complete high school	140	100 (71.4)		64 (45.7)	
University	197	148 (75.1)		72 (36.5)	

Mother's occupation			0.84		0.34
Housewife	398	300 (75.4)		167 (42)	
Not housewife	62	46 (74.2)		22 (35.5)	

Mother's education			0.72		0.54
Under complete high school	134	102 (76.1)		60 (44.8)	
Complete high school	194	140 (72.2)		85 (43.8)	
University	90	67 (74.4)		34 (37.8)	

^*∗*^Pearson *χ*^2^ test.

**Table 3:** Results of univariate logistic regression analysis of associations by dentition type.

Variables	Primary dentition		Permanent dentition	
	OR (95% CI)	*p* value	OR (95% CI)	*p* value
Age (year)				
7–8	1^†^	—	1^†^	—
9–10	0.3 (0.127–0.709)	0.006	1.8 (1.103–2.932)	0.019
11–12	0.049 (0.021–0.111)	<0.001	3.675 (2.219–6.088)	<0.001

Sex				
Boy	1^†^		1^†^	
Girl	0.84 (0.779–1.818)	0.42	1.47 (1.012–2.135)	0.04^*∗*^

Resident area				
Urban	1^†^		1^†^	
Rural	1.06 (0.677–1.609)	0.79	2.49 (1.676–3.706)	<0.001^*∗*^

Father's occupation				
Employee	1^†^		1^†^	
Not employee	1.40 (0.906–2.183)	0.12	1.53 (1.074–2.254)	0.02^*∗*^

Mother's occupation				
Housewife	1^†^		1^†^	
Not housewife	0.93 (0.509–1.733)	0.84	0.76 (0.436–1.328)	0.33

Father's education level				
Under complete high school	1^†^		1^†^	
Complete high school	0.81 (0.490–1.349)	0.42	0.80 (0.465–1.384)	0.42
University	0.91 (0.493–1.696)	o.77	0.54 (0.326–0.924)	0.24

Mother's education level				
Under complete high school	1^†^		1^†^	
Complete high school	0.86 (0.465–1.595)	0.63	0.96 (0.617–1.498)	0.86
University	1.04 (0.575–1.879)	0.89	0.74 (0.434–1.292)	0.29

^†^Reference group.

## Data Availability

The data used to support the findings of this study are available from the corresponding author upon request.
